# Cardiac output monitoring in cirrhotic patients: EV1000 versus pulmonary artery catheter - preliminary data

**DOI:** 10.1186/cc10826

**Published:** 2012-03-20

**Authors:** G Costa, T Cecconet, D Baron, G Serena, P Chiarandini, L Pompei, L Vetrugno, G Della Rocca

**Affiliations:** 1 University of Udine, Italy

## Introduction

The EV1000 platform, a new calibrated device for intermittent and continuous cardiac output monitoring, has recently been introduced into clinical practice [1]. This study aims to assess the level of agreement between intermittent and continuous cardiac output obtained from VolumeView (ICOvv and CCOvv) connected to the EV1000 platform (Edwards Lifesciences, Irvine, CA, USA) and intermittent (ICOvig) and continuous cardiac output (CCOvig) obtained using an advanced pulmonary artery catheter (PAC) connected to the Vigilance System (Edwards Lifesciences) in cirrhotic patients undergoing liver transplantation.

## Methods

Seven consecutive patients (seven male, zero female), mean age 56.5 (± 12) years, were enrolled into the study. ICO data were obtained from the two devices after ICU admission (T0) and after 12 (T12) and 24 hours (T24). CCOvig and CCOvv were recorded every hour from T0 up to 48 hours after ICU admission. Agreement and precision between CO values were evaluated with Bland-Altman analysis. The percentage error (PE) was calculated as 2SD/mean CO [2].

## Results

Twenty-one ICO data pairs were compared. Two patients were excluded from CCO data analysis for technical reasons. A total 240 CCO data pairs from five patients were analysed. Data yielded were analysed as total and for CO values lower and higher than 8 l/minute (Table 1).

**Table 1:** Bias, 2SD and PE for all data pairs and for CO higher and lower than 8 l/minute

	Bias	2SD	PE (%)
ICO			
Total	-0.89	3.6	49
<8	-1.37	2.4	40
>8	-0.05	4.8	52
CCO			
Total	-0.83	4.88	63
<8	-1.8	2.4	37
>8	2.07	5.64	47

## Conclusion

These data, even if very preliminary, showed low agreement and high PE either for intermittent and continuous CO obtained from the VolumeView. However, for CO data lower than 8 l/minute the PE was improved.
